# Supplementary material for: Are Gluten-Free Diets More Nutritious? An Evaluation of Self-Selected and Recommended Gluten-Free and Gluten-Containing Dietary Patterns
Source: Nutrients. 2018 Dec 3;10(12):1881. doi: 10.3390/nu10121881 (PMC6317051; doi:10.3390/nu10121881)
Supplement: Supplementary file 1 [file nutrients-10-01881-s001.pdf]

Supplementary Table S1: MyPlate seven-day gluten containing menus with gluten free modifications

DAY 1		
	**Gluten Containing**	**Gluten Free**
**Breakfast**	uncooked oatmeal<br>fat-free milk<br>raisins<br>brown sugar<br>orange juice	uncooked oatmeal<br>fat-free milk<br>raisins<br>brown sugar<br>orange juice
**Lunch**	tortilla chips<br>cooked ground turkey<br>corn oil<br>kidney beans<br>low-fat cheddar cheese<br>chopped lettuce<br>avocado<br>lime juice<br>salsa<br>coffee	tortilla chips<br>cooked ground turkey<br>corn oil<br>kidney beans<br>low-fat cheddar cheese<br>chopped lettuce<br>avocado<br>lime juice<br>salsa<br>coffee
**Dinner**	dry lasagna noodles (two pieces)<br>cooked spinach<br>ricotta cheese<br>part-skim mozzarella cheese<br>tomato sauce<br>whole wheat roll<br>tub margarine<br>fat-free milk	gluten free rice pasta<br>cooked spinach<br>ricotta cheese<br>part-skim mozzarella cheese<br>tomato sauce<br>gluten-free multigrain rolls<br>tub margarine<br>fat-free milk
**Snacks**	raisins<br>unsalted almonds	raisins<br>unsalted almonds

DAY 2		
	**Gluten Containing**	**Gluten Free**
**Breakfast**	flour tortilla<br>scrambled egg<br>black beans<br>salsa<br>large grapefruit<br>tea	gluten-free plain tortilla<br>scrambled egg<br>black beans<br>salsa<br>large grapefruit<br>tea
**Lunch**	small whole grain hoagie bun<br>lean roast beef<br>part-skim mozzarella cheese<br>tomato<br>mushrooms<br>canola oil<br>mustard<br>potato wedges	gluten free whole grain sandwich roll<br>lean roast beef<br>part-skim mozzarella cheese<br>tomato<br>mushrooms<br>canola oil<br>mustard<br>potato wedges

	corn oil<br>ketchup<br>fat-free milk	corn oil<br>ketchup<br>fat-free milk
**Dinner**	salmon filet<br>olive oil<br>lemon juice<br>cooked beat greens<br>canola oil<br>quinoa<br>slivered almonds<br>fat-free milk	salmon filet<br>olive oil<br>lemon juice<br>cooked beat greens<br>canola oil<br>quinoa<br>slivered almonds<br>fat-free milk
**Snacks**	cantaloupe balls	cantaloupe balls

**DAY 3**		
	**Gluten Containing**	**Gluten Free**
**Breakfast**	medium banana<br>fat-free milk<br>whole wheat toast<br>tub margarine<br>Multigrain Cheerios<br>prune juice	medium banana<br>fat-free milk<br>gluten-free 7-grain bread<br>tub margarine<br>gluten-free Cereal<br>prune juice
**Lunch**	rye bread<br>tuna<br>mayonnaise<br>chopped celery<br>shredded lettuce<br>medium peach<br>fat-free milk	gluten-free whole grain rye style bread<br>tuna<br>mayonnaise<br>chopped celery<br>shredded lettuce<br>medium peach<br>fat-free milk
**Dinner**	cooked chicken breast<br>large sweet potato, roasted<br>succotash (lima beans and corn)<br>tub margarine<br>whole wheat roll<br>tub margarine<br>coffee	cooked chicken breast<br>large sweet potato, roasted<br>succotash (lima beans and corn)<br>tub margarine<br>gluten-free multigrain rolls<br>tub margarine<br>coffee
**Snacks**	dried apricots<br>flavored yogurt (chocolate)	dried apricots<br>flavored yogurt (chocolate)

**DAY 4**		
	**Gluten Containing**	**Gluten Free**

**Breakfast**	whole wheat english muffin<br>all-fruit preserves<br>hard-cooked egg<br>tea	brown rice english muffin<br>all-fruit preserves<br>hard-cooked egg<br>tea
**Lunch**	chunky vegetable soup with pasta<br>white beans<br>saltine crackers<br>celery sticks<br>fat-free milk	gluten-free veggie noodle soup<br>white beans<br>gluten-free table crackers<br>celery sticks<br>fat-free milk
**Dinner**	dry rigatoni pasta<br>cooked ground beef (95% lean)<br>corn oil<br>tomato sauce<br>grated parmesan cheese<br>raw spinach leaves<br>tangerine sections<br>chopped walnuts<br>oil and vinegar dressing<br>tea	gluten-free fusilli<br>cooked ground beef (95% lean)<br>corn oil<br>tomato sauce<br>grated parmesan cheese<br>raw spinach leaves<br>tangerine sections<br>chopped walnuts<br>oil and vinegar dressing<br>tea
**Snacks**	nonfat fruit yogurt	nonfat fruit yogurt

**DAY 5**		
	**Gluten Containing**	**Gluten Free**
**Breakfast**	shredded wheat<br>sliced banana<br>fat-free milk<br>whole wheat toast<br>all-fruit preserves<br>fat-free chocolate milk	shredded wheat<br>sliced banana<br>fat-free milk<br>gluten-free 7-grain bread<br>all-fruit preserves<br>fat-free chocolate milk
**Lunch**	whole wheat pita bread<br>roasted turkey<br>tomato<br>shredded lettuce<br>mustard<br>mayonnaise<br>grapes<br>tomato juice	gluten-free, wheat-free wraps ivory teff<br>roasted turkey<br>tomato<br>shredded lettuce<br>mustard<br>mayonnaise<br>grapes<br>tomato juice
**Dinner**	broiled beef steak<br>mashed potatoes made with milk<br>tub margarine<br>cooked green beans<br>tub margarine<br>honey<br>whole wheat roll	broiled beef steak<br>mashed potatoes made with milk<br>tub margarine<br>cooked green beans<br>tub margarine<br>honey<br>gluten-free multigrain rolls

	tub margarine<br>frozen yogurt (chocolate)<br>sliced strawberries<br>fat-free milk	tub margarine<br>frozen yogurt (chocolate)<br>sliced strawberries<br>fat-free milk
**Snacks**	frozen yogurt (chocolate)	frozen yogurt (chocolate)

**DAY 6**		
	**Gluten Containing**	**Gluten Free**
**Breakfast**	whole wheat bread<br>fat-free milk<br>egg<br>tub margarine<br>pancake syrup<br>large grapefruit<br>fat-free milk	gluten-free mountain white bread<br>fat-free milk<br>egg<br>tub margarine<br>pancake syrup<br>large grapefruit<br>fat-free milk
**Lunch**	cooked kidney beans<br>navy beans<br>black beans<br>tomato sauce<br>chopped onion<br>chopped jalapeno peppers<br>canola oil<br>cheese sauce<br>large baked potato<br>cantaloupe<br>coffee	cooked kidney beans<br>navy beans<br>black beans<br>tomato sauce<br>chopped onion<br>chopped jalapeno peppers<br>canola oil<br>cheese sauce<br>large baked potato<br>cantaloupe<br>coffee
**Dinner**	cheese pizza, thin crust<br>lean ham<br>pineapple<br>mushrooms<br>safflower oil<br>romaine<br>spinach<br>oil and vinegar dressing<br>fat-free milk	three cheese pizza (half a pizza)<br>lean ham<br>pineapple<br>mushrooms<br>safflower oil<br>romaine<br>spinach<br>oil and vinegar dressing<br>fat-free milk
**Snacks**	hummus<br>whole wheat crackers	hummus<br>gluten-free multigrain crackers

**DAY 7**		
	**Gluten Containing**	**Gluten Free**
**Breakfast**	large (7") buckwheat pancakes<br>pancake syrup<br>sliced strawberries<br>orange juice	large (7") buckwheat pancakes<br>pancake syrup<br>sliced strawberries<br>orange juice

**Lunch**	canned clams<br>small potato<br>chopped onion<br>chopped celery<br>evaporated milk<br>fat-free milk<br>bacon<br>white flour<br>whole wheat crackers<br>medium orange<br>fat-free milk	canned clams<br>small potato<br>chopped onion<br>chopped celery<br>evaporated milk<br>fat-free milk<br>bacon<br>white rice flour<br>gluten-free multigrain crackers<br>medium orange<br>fat-free milk
**Dinner**	firm tofu<br>chopped chinese cabbage<br>sliced bamboo shoots<br>chopped sweet red peppers<br>chopped sweet green peppers<br>corn oil<br>cooked brown rice<br>honeydew melon<br>plain fat-free yogurt<br>tea	firm tofu<br>chopped chinese cabbage<br>sliced bamboo shoots<br>chopped sweet red peppers<br>chopped sweet green peppers<br>corn oil<br>cooked brown rice<br>honeydew melon<br>plain fat-free yogurt<br>tea
**Snacks**	large banana<br>peanut butter<br>nonfat fruit yogurt	large banana<br>peanut butter<br>nonfat fruit yogurt
